# Transcriptomic profiling of rose flower under treatment of various phytohormones and plant growth regulators

**DOI:** 10.1038/s41597-022-01800-w

**Published:** 2022-11-03

**Authors:** Xintong Liu, Jie Wu, Fangfang Ji, Xiaoqian Cao, Qingcui Zhao, Chenxia Cheng, Nan Ma, Xiaofeng Zhou, Zhao Zhang

**Affiliations:** 1 Beijing Key Laboratory of Development and Quality Control of Ornamental Crops, Department of Ornamental Horticulture, College of Horticulture, China Agricultural University, Beijing, 100193 China; 2 Key Laboratory for Quality Regulation of Tropical Horticultural Crops of Hainan Province, School of Horticulture, Hainan University, Haikou, China

**Keywords:** Transcriptomics, Gene expression

## Abstract

Rose is one of the most important ornamental plants, accounting for one-third of the world’s fresh cut flower market. The vase life refers to the period of a cut flower retaining its appearance in a vase. During this period, the rose was subjected to a variety of abiotic and biotic stresses, resulting in a reduction in the life of cut flowers. Numerous studies have been carried out on cut rose, which proves the effects of various plant hormones on post-harvest dehydration, petal senescence and abscission, disease and vase life of cut rose flowers. In addition, the natural or synthetic hormones or its inhibitor have been successfully used in cut flower preservatives to extend the vase life of rose. However, there is still a lack of systematic and in-depth research on the expression of rose genes related to plant hormone response. Here we analyzed the gene expression changes of the rose flower under treatment of 11 different plant hormones or its inhibitors in order to provide reference for rose studies.

## Abstract (summary)

**Table:** 

Measurement(s)	Transcripts expression profile
Technology Type(s)	Illumina HiSeq
Factor Type(s)	phytohormones
Sample Characteristic - Organism	Rosa hybrida

## Background & Summary

Rose (*Rosa* sp.) is the most popular flower crop in the world. With its long history of cultivation, the rose has been endowed with cultural connotations in both the eastern and western world. In 2016, the worldwide turnover of cut roses was 4.96 billion euros, accounting for 29.7% of total cut flowers, which was the largest in flower crops (AIPH, 2016).

The vase life refers to the duration of a cut flower retaining its appearance in a vase. It is therefore the most important trait to the ornamental crops that are used as a cut flower, including rose. During the early vase life period, flowers open and appeal flavor, later they start the senescence, dehydration and abscission of petals. In addition, cut flowers are also subject to postharvest diseases, such as gray mold disease caused by the necrotrophic fungus *Botrytis cinerea*. All these physiological changes occur at a specific stage and in a highly synchronized manner, involving the balance of phytohormones in the flower and up- or down-regulation of numerous genes in various hormones pathways.

Therefore, the effects of plant hormones that extend vase life are major component of floriculture research and have been studied at the physiological and biochemical levels for several decades. Many of chemicals involved in plant hormones and their inhibitors have been added into the preservatives to improve the post-harvesting fresh keeping and vase life of cut rose. In rose, a number of studies have reported that flower opening, petal senescence, dehydration and abscission can be affected by abscisic acid (ABA), cytokinins (CKs), ethylene (ET) and gibberellins (GAs)^1–8^. In addition, recently, role of brassinosteroids (BRs), jasmonic acid (JA) and ET in rose petal defense against *B. cinerea* infection have been reported^9,10^.

However, the global gene expression pattern of rose flowers behind hormonal treatment has not been well-studied yet. To date, the only involved transcriptome data is screening of ethylene responsive genes from rose flowers (SRA045958), which is currently presented in the NCBI Sequence Read Archive (SRA)^11^.

The information on the molecular mechanism of phytohormones regulating flower traits remains scarce due to the lack of transcriptome. Therefore, transcriptome data from rose petals after different hormone treatments will be useful for studying the expression patterns of hormone-related genes and excavating key genes that regulate flower traits. Using RNA-seq, we recently investigated the transcriptomic dynamics of rose flower under the treatment of eleven natural or synthetic hormones, including auxin, BRs, CKs, GAs, ABA, JA, salicylic acid (SA), ethylene (ET) as well as ethylene inhibitors. We obtained approximately 240 Gb data and dissected the transcriptional network with the aim of exploring the transcriptional variation of rose responses towards those plant hormones. Our data will be useful to all those working with the analysis of rose gene expression.

## Methods

### Plant materials

*Rosa hybrida* ‘Samantha’, a classic hybrid tea rose cultivar and frequently used for cut flowers, has a red colour and mild fragrance. The ‘Samantha’ plant was grown under a plastic cover in Changping District (40°139 N, 116°129E), Beijing, China. In Spring of 2017, cut flower samples were harvested at developmental stage 2 of flower opening^1^.

### Exogenous phytohormone treatment

Flowering rose stems were cut into lengths of 30 cm and placed in aqueous solutions of 1-naphthaleneacetic acid (NAA), 2,4-dichlorophenoxyacetic acid (2,4-D), 2,4-epibrassinolide (BR), 6-benzylaminopurine (6-BA), ABA, gibberellic acid 3 (GA3), JA, SA, as well as ethylene inhibitor AgNO_3_, respectively, for 24 h under the controlled conditions of 22 °C with 30% to 40% relative humidity and 16 h/8 h day/night periods. ‘Samantha’ treated with deionized water were used as the control. For ethylene (ET) and 1-methylcyclopropene (1-MCP) treatment, rose flowers were exposed to ethylene, 1-MCP, or regular air as the control, for 24 h and 1 M NaOH was used to absorb CO_2_ released by respiration. The detailed information and final concentration of chemical agents were listed in Table 1. For each treatment, three replicates were harvested and 4 flowers were randomly collected for each replicate.

**Table 1:** The information of exogenous phytohormone in vase treatments.

The name of chemical agents	Abbreviation	Specie of phytohormone	Final concentration
1-Naphthaleneacetic acid	NAA	Auxin	100 µM
2, 4-Dichlorophenoxy acetic acid	2,4-D	Auxin	100 µM
2, 4-Epibrassinolide	BR	Brassinosteroid	5.0 µM
6-Benzylamino purine	6-BA	Cytokinin	100 µM
(+)-Abscisic Acid	ABA	Abscisic acid	100 µM
Gibberellic acid 3	GA3	Gibberellin	80 µM
(±)-Jasmonic acid	JA	Jasmonic acid	50 µM
Salicylic acid	SA	Salicylic acid	100 µM
Ethylene	ET	Ethylene	10 μL/L
1-Methylcyclopropene	1-MCP	Ethylene inhibitor	2 μL/L
Silver nitrate	AgNO_3_	Ethylene inhibitor	80 µM

### RNA extraction, library construction, and Illumina sequencing

Total RNA was extracted from the outer layer of rose petals using the hot borate reagent following the previous description^3^. The quality of the RNA was verified by agarose gel electrophoresis, NanoDrop 2000 spectrophotometers (Thermo Fisher Scientific) and Agilent Technologies 2100 Bio-analyzer, all samples QC results were shown in Table 2. The libraries were sequenced on the Illumina HiSeq™ 2500 system (Illumina Inc., San Diego, CA), according to the manufacturer’s instructions. Illumina sequencing was conducted at Novogene, Beijing, China.

**Table 2:** RNA QC Results Summary.

Sample name	OD260/280	OD260/230	RIN
H2O-1	1.859	2.19	6.6
H2O-2	1.833	2.373	6.5
H2O-3	1.858	2.411	7.2
NAA-1	1.759	1.723	7.7
NAA-2	1.846	2.099	7.1
NAA-3	1.978	2.067	6.8
2,4-D-1	1.73	2.247	6.3
2,4-D-2	1.756	1.8	7.5
2,4-D-3	1.989	2.08	7.3
BR-1	1.843	2.025	6.5
BR-2	1.985	2.015	6.9
BR-3	1.987	2.429	6.3
6-BA-1	1.8	2.359	6.5
6-BA-2	1.86	1.839	6.4
6-BA-3	1.791	1.983	6.3
ABA-1	1.795	1.826	7.1
ABA-2	1.848	2.2	6.4
ABA-3	1.84	2.035	6.6
GA3-1	1.877	2.0	7.5
GA3-2	1.879	2.192	7.1
GA3-3	1.776	2.177	6.5
JA-1	1.886	2.357	7.2
JA-2	1.977	2.177	7.0
JA-3	1.975	2.286	7.1
SA-1	1.96	2.202	6.5
SA-2	1.75	2.167	6.9
SA-3	1.883	2.164	6.8
ET-CK-1	1.711	2.119	6.2
ET-CK-2	1.805	2.124	6.5
ET-CK-3	1.784	2.141	7.3
ET-1	1.733	2.011	7.6
ET-2	1.897	2.26	7.1
ET-3	1.889	2.092	8.0
1-MCP-1	1.721	1.52	6.9
1-MCP-2	1.871	2.304	7.2
1-MCP-3	1.888	2.182	6.7
AgNO_3_-1	1.89	2.013	7.4
AgNO_3_-2	2	2.116	7.7
AgNO_3_-3	1.928	1.975	8.1

## Data Records

Our 39 raw data of RNA-seq were deposited into the NCBI database at Sequence Read Archive (SRA) with the accession number PRJNA522664^12^.

Average gene expression (fragments per kb per million reads, FPKM) information of each experiment was deposited in Gene Expression Omnibus (GEO) in NCBI, number GSE140696^13^.

## Technical Validation

**Fig. 1:**
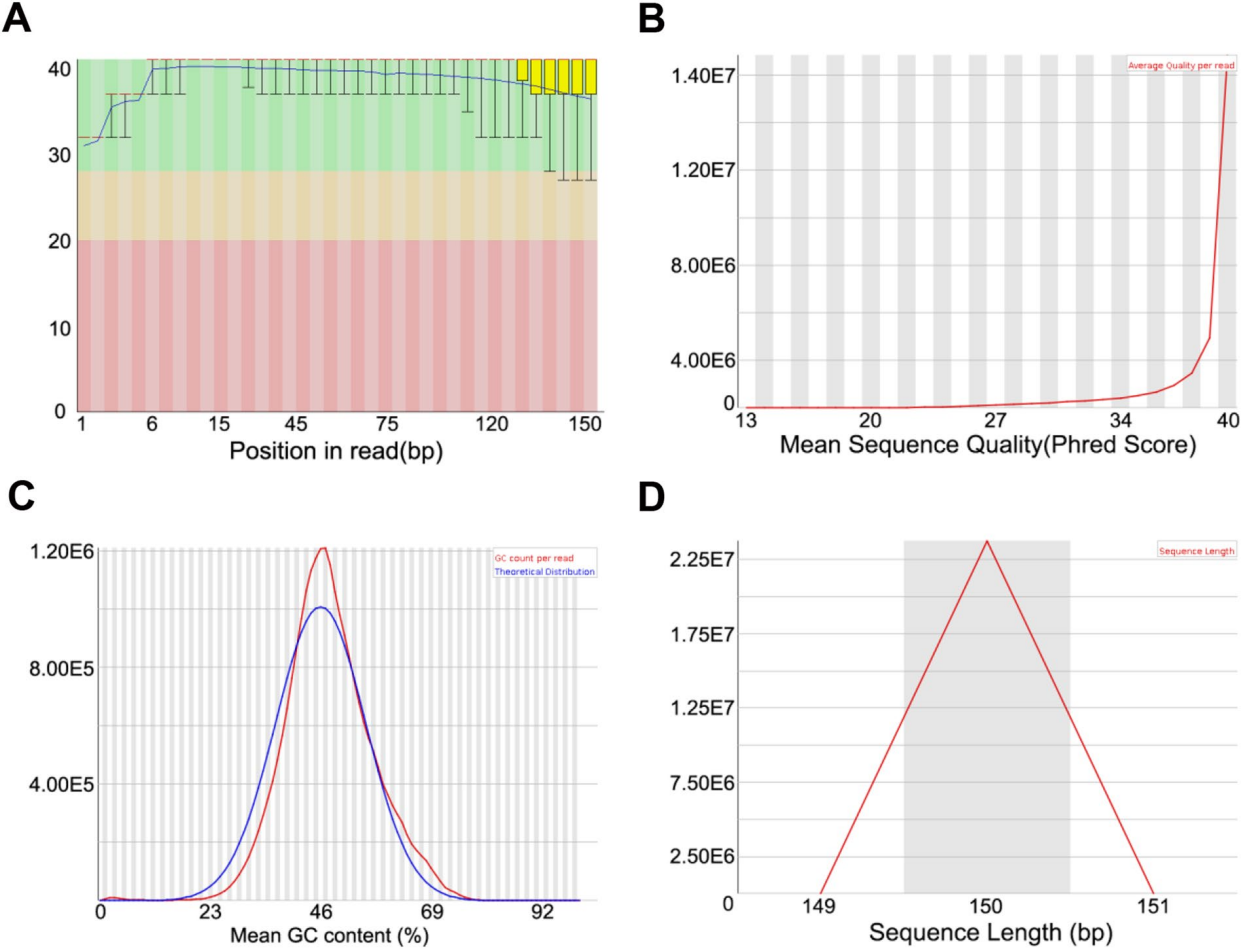
Quality control result of BR-3 clean data. (**A**) Quality score of per position in read. (**B**) Quality score of mean sequence. (**C**) GC content distribution. (**D**) Sequence length distribution.

**Fig. 2:**
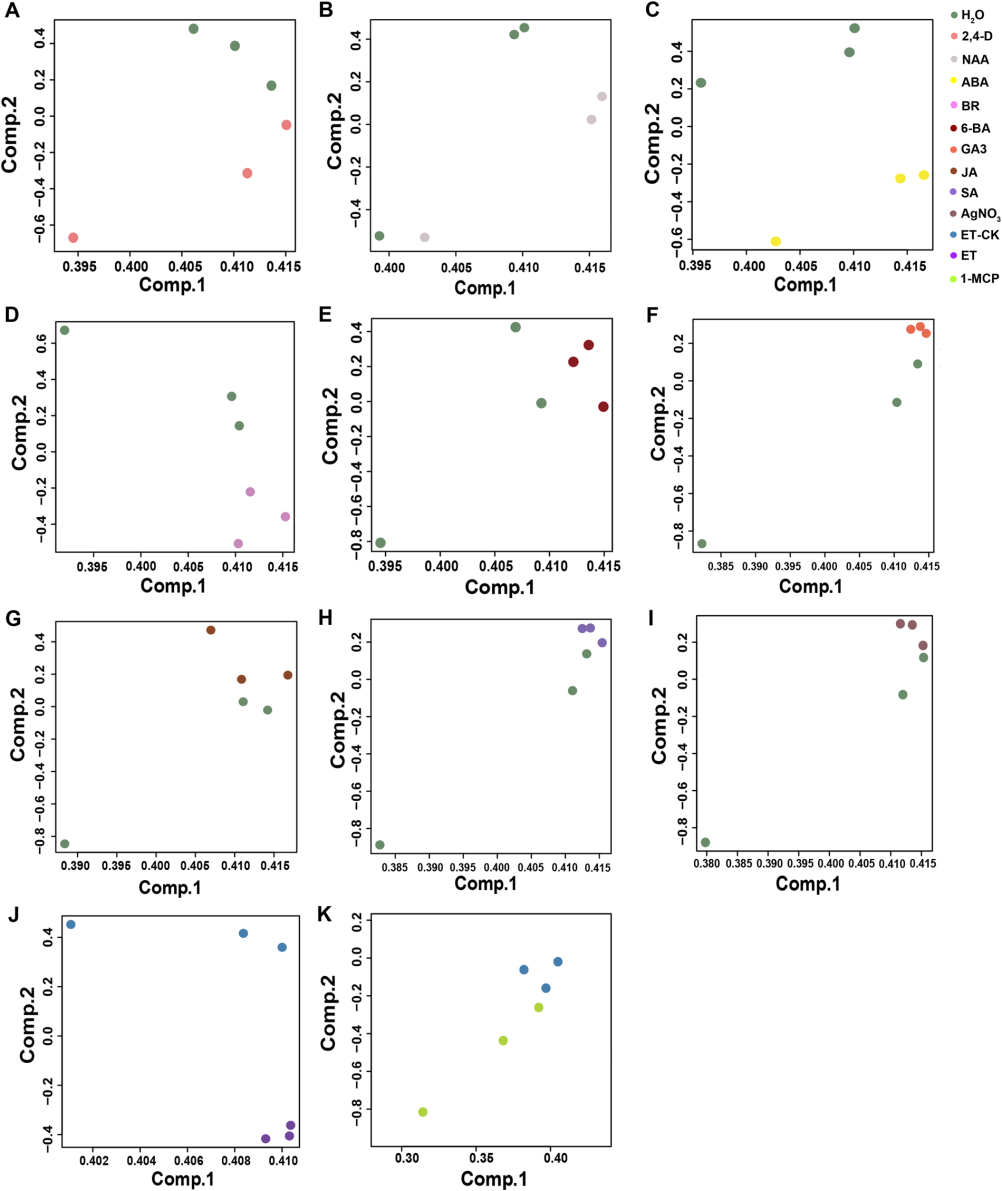
The principal components analysis (PCA) of RNA-seq data following hormone treatments. (**A**–**I**). represented the PCA of 2,4-D, NAA, ABA, BR, 6-BA, GA3, JA, SA and AgNO_3_ compared with mock treatment (H_2_O). (**J**) and (**K**) represented the PCA of ET and 1-MCP compared with air (ET-CK).

Our 39 raw data were achieved on Illumina HiSeq. The raw data was cleaned by removing the adaptor sequences, reads containing N > 10% (N represents the base cannot be determined), as well as low quality (Qscore <  = 5) base which is over 50% of the total base (Table 3)^14^. FastQC was used to test the quality of 78 paired-end clean data^15^. We have shown the quality control results of BR-3 clean data as an illustration (Fig. 1). The FPKM (fragments per kb per million reads) of 39 samples representing different treatments were subjected to principal component analysis (PCA), and the clear separation between the treatment and mock was detected (Fig. 2).

**Table 3:** Data Quality Summary.

Sample_name	Raw_base (bp)	Clean_base (bp)	Raw reads	Clean reads	Effective rate (%)	Q20	Q30	GC content (%)
H2O-1	6715943400	6648069900	44772956	44320466	98.99	99.47	91.89	46
H2O-2	6794421300	6474099000	45296142	43160660	95.29	99.67	92.65	47
H2O-3	6208617900	6104910000	41390786	40699400	98.33	99.49	91.89	46.5
NAA-1	6352380600	6279223800	42349204	41861492	98.85	99.41	91.74	47
NAA-2	6248699400	6161616600	41657996	41077444	98.61	99.54	92.05	47
NAA-3	6292710900	6216902700	41951406	41446018	98.80	99.65	93.25	46
2, 4-D-1	6141073800	6010109100	40940492	40067394	97.87	99.54	92.75	46
2, 4-D-2	5737084500	5660827200	38247230	37738848	98.67	99.44	91.29	47
2, 4-D-3	7018744800	6884462400	46791632	45896416	98.09	99.34	91.37	46
BR-1	6426085200	6285365700	42840568	41902438	97.81	99.53	92.26	46
BR-2	6138147600	6041525700	40920984	40276838	98.43	99.48	92.01	46
BR-3	7274191500	7115562300	48494610	47437082	97.82	99.60	93.31	47
6-BA-1	6110882400	5885013600	40739216	39233424	96.30	99.61	93.09	46
6-BA-2	5746281900	5627598000	38308546	37517320	97.93	99.59	92.79	47
6-BA-3	5562834300	5495274600	37085562	36635164	98.79	99.53	92.06	46.5
ABA-1	6648969000	6559461300	44326460	43729742	98.65	99.45	91.84	47
ABA-2	7331724000	7209520500	48878160	43729742	89.47	99.39	91.05	46.5
ABA-3	6873168000	6733405800	45821120	44889372	97.97	99.45	91.85	46
GA3-1	7228373700	7149807600	48189158	47665384	98.91	99.51	92.31	46
GA3-2	5687607600	5638328100	37917384	37588854	99.13	99.49	92.04	46.5
GA3-3	5946696000	5891745300	39644640	39278302	99.08	99.52	91.80	46
JA-1	6626836800	6517555200	44178912	43450368	98.35	99.41	91.62	47
JA-2	5917674300	5765472000	39451162	38436480	97.43	99.71	93.50	46
JA-3	6022036200	5938326000	40146908	39588840	98.61	99.52	90.50	46.5
SA-1	6053166600	5962658100	40354444	39751054	98.50	99.55	92.04	46
SA-2	5785421100	5706178200	38569474	38041188	98.63	99.64	92.62	46
SA-3	6465527400	6383856000	43103516	42559040	98.74	99.59	92.69	46
ET-CK-1	7329915600	7182532800	48866104	47883552	97.99	99.68	93.39	46
ET-CK-2	6057890700	5956388400	40385938	39709256	98.32	99.50	91.83	46
ET-CK-3	7039669800	6896620200	46931132	45977468	97.97	99.53	92.33	46
ET-1	7203534600	7115364900	48023564	47435766	98.78	99.63	93.08	46
ET-2	6734019600	6604808700	44893464	44032058	98.08	99.64	92.74	46
ET-3	7487982600	7285686300	49919884	48571242	97.30	99.63	92.90	46
1-MCP-1	6570133800	6514792800	43800892	43431952	99.16	99.47	91.99	48
1-MCP-2	6679282800	6591700500	44528552	43944670	98.69	99.68	94.05	46
1-MCP-3	6255930600	6074547600	41706204	40496984	97.10	99.51	91.73	47
AgNO3-1	6720336300	6563929800	44802242	43759532	97.67	99.57	92.41	46.5
AgNO3-2	6396397200	6308241900	42642648	42054946	98.62	99.54	92.38	46.5
AgNO3-3	6068345400	5977800600	40455636	39852004	98.51	99.64	92.26	46

All data aboved were counted for read1 + read2. Raw bases: (Raw reads) * (sequence length). Clean bases: (Clean reads) * (sequence length). For paired-end sequencing like PE150, sequencing length equals 150, otherwise it equals 50 for sequencing like SE50. Effective Rate (%): (Clean reads/Raw reads) *100% Error rate: base error rate Q20, Q30: (Base count value > 20 or 30) / (Total base count). GC content: (G & C base count)/(Total base count).

Clean reads were mapped to reference genome *Rosa chinensis* ‘Old blash’ (RchiOBHm-V2, GCF_002994745.1)^16^ by default parameters of Tophat2(version 2.1.1). Then use Cufflinks (version 2.2.0) to generate transcriptome with the Tophat2 (version 2.1.1) resulting alignment files, the assemblies were merged with the Cuffmerge, which is included in the Cufflinks package. These merged results provide a uniform basis for calculating gene and transcript expression. Then the merged assembles were provided to Cuffdiff, which calculated expression levels and tested the statistical significance of observed changes.

**Fig. 3:**
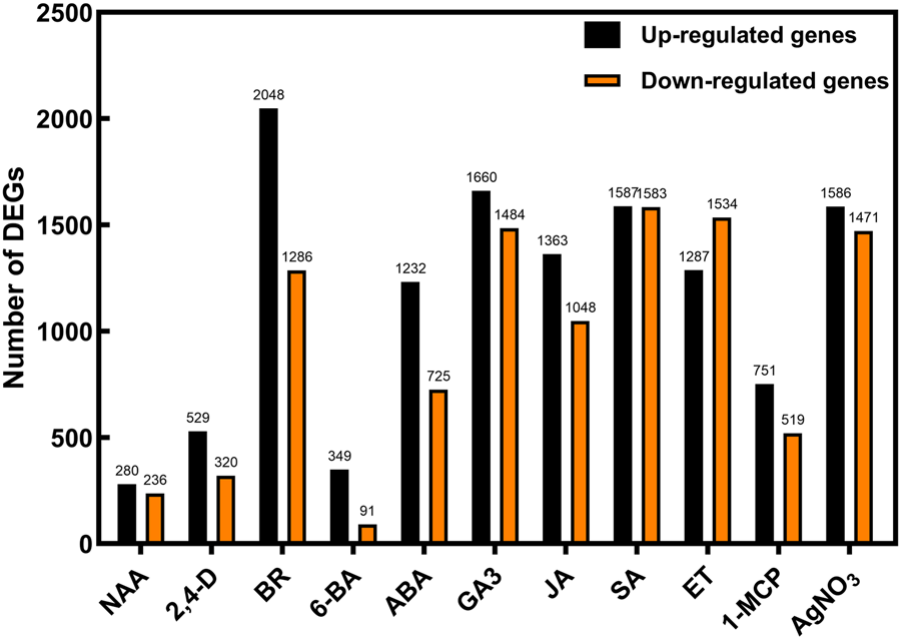
The number of different expression genes (DEGs) in rose petals under exogenous phytohormone treatments. The DEGs were determined with |log2 fold change (FC)| ≥ 0.5, and an adjusted *P*-value < 0.05.

Average gene expression information of each experiment was deposited in Gene Expression Omnibus (GEO) in NCBI, number GSE140696. The differentially expressed genes (DEGs) were analyzed by DESeq (Anders *et al*., 2013) and defined as genes with |log2 fold change (FC)| ≥ 0.5, and an adjusted *P*-value < 0.05. The number of DEGs for 11 treatments compared with the control, was shown in Fig. 3.

**Fig. 4:**
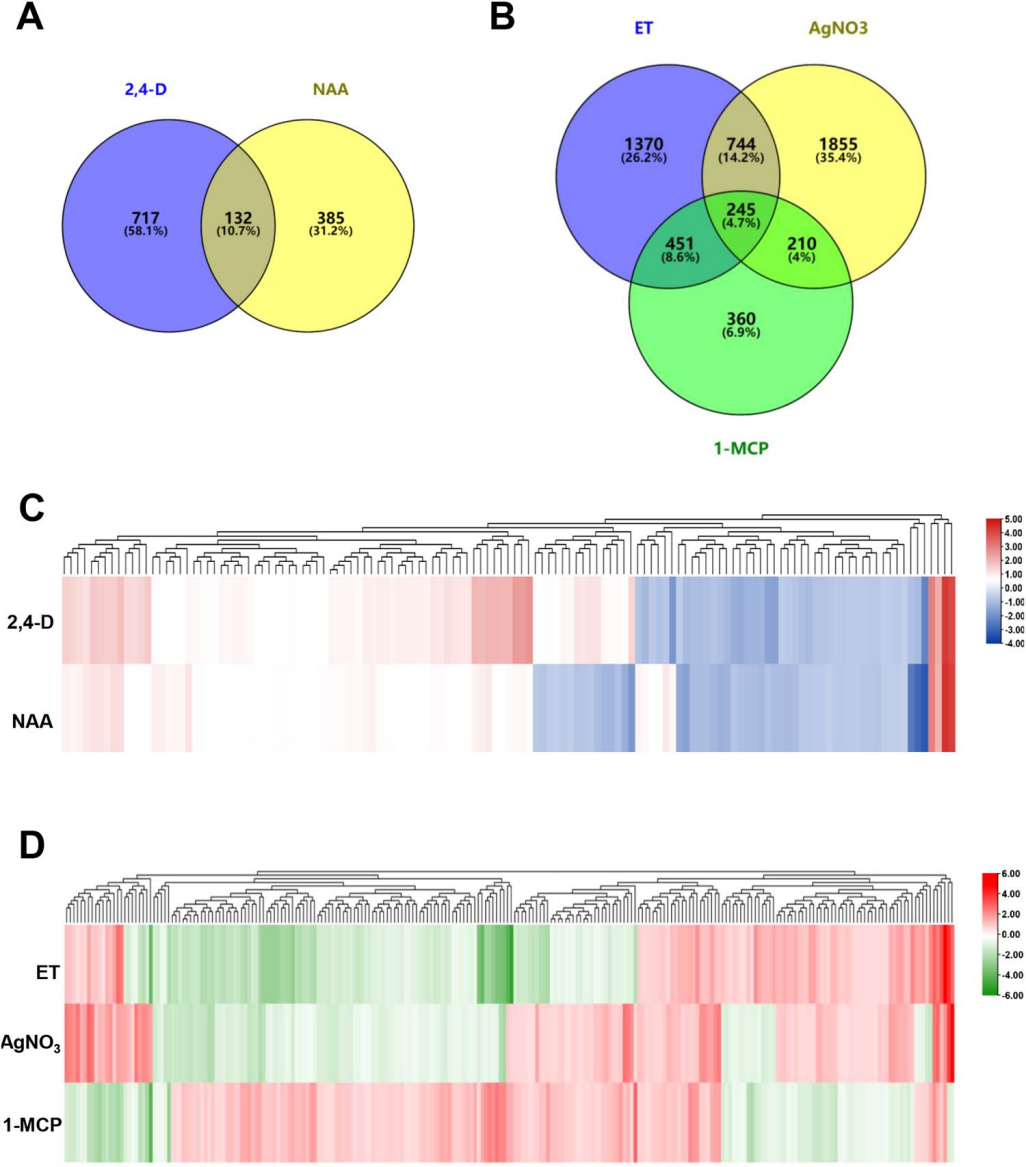
The DEGs among auxin related hormone (2,4-D and NAA), ethylene and its inhibitors (AgNO_3_ and 1-MCP). (**A**). Venn diagram depicted the number and overlap DEGs from 2,4-D- and NAA- treatments. (**B**). Venn diagram depicted the number and overlap DEGs from ET-, AgNO3- and 1-MCP- treatments. (**C**). The heatmap of overlap DEGs by hierarchical cluster analysis among 2,4-D- and NAA- treatments. (**D**). The heatmap of overlap DEGs by hierarchical cluster analysis among ET-, AgNO3- and 1-MCP- treatments.

In addition, we have analyzed the DEGs under auxin-related plant growth regulators (2,4-D and NAA) and ethylene-related chemicals (ET, 1-MCP and AgNO_3_). The results showed that 132 overlapped DEGs were identified under 2,4-D and NAA treatments (Fig. 4A). Among the 132 DEGs, 84.09% showed a similar expression pattern under the two different auxin-related plant growth regulators (Fig. 4C). In ET-, 1-MCP- and AgNO_3_ treatments, there were 245 overlapped DEGs were screened out (Fig. 4B). Although both 1-MCP and AgNO_3_ are the inhibitors of ET, they work in different ways. We have identified that 81.3% and 26.1% of the 245 DEGs played an opposite expression pattern in 1-MCP and AgNO_3_ treatment compared with it in ET treatment, respectively (Fig. 4D).

## Data Availability

The following software and versions were used for quality control and data processing: 1. FastQC: https://www.bioinformatics.babraham.ac.uk/projects/fastqc (version 0.11.9). 2. TopHat2: http://ccb.jhu.edu/software/tophat/index.shtml (version 2.1.1). 3. Cufflinks: http://cole-trapnell-lab.github.io/cufflinks(version 2.2.0).

## References

[1] Ma N, Cai L, Lu W, Tan H, Gao J (2005). Exogenous ethylene influences flower opening of cut roses (*Rosa hybrida*) by regulating the genes encoding ethylene biosynthesis enzymes. Sci China C Life Sci..

[2] Tan H (2006). Ethylene-influenced flower opening and expression of genes encoding ETRs, CTRs, and EIN3s in two cut rose cultivars. Postharvest Biol Technol..

[3] Wu L (2017). An Ethylene-induced regulatory module delays flower senescence by regulating cytokinin content. Plant Physiol..

[4] Xue J (2008). Expression of ethylene biosynthetic and receptor genes in rose floral tissues during ethylene-enhanced flower opening. J Exp Bot..

[5] Ma N (2008). Rh-PIP2; 1, a rose aquaporin gene, is involved in ethylene-regulated petal expansion. Plant Physiol..

[6] Ma N (2018). Petal senescence: a hormone view. J Exp Bot..

[7] Lü P (2014). RhHB1 mediates the antagonism of gibberellins to ABA and ethylene during rose (*Rosa hybrida*) petal senescence. Plant J..

[8] Luo J (2013). A *DELLA* gene, *RhGAI1*, is a direct target of EIN3 and mediates ethylene-regulated rose petal cell expansion via repressing the expression of *RhCesA2*. J Exp Bot..

[9] Liu X (2018). Comparative RNA-Seq analysis reveals a critical role for brassinosteroids in rose (*Rosa hybrida*) petal defense against *Botrytis cinerea* infection. BMC Genet..

[10] Cao X (2019). A detached petal disc assay and virus-induced gene silencing facilitate the study of *Botrytis cinerea* resistance in rose flowers. Hortic Res.

[11] Pei H (2013). An NAC transcription factor controls ethylene-regulated cell expansion in flower petals. Plant Physiol..

[12] (2019). NCBI database at Sequence Read Archive.

[13] Liu X, Wu J (2019). Gene Expression Omnibus.

[14] Jiang L (2011). Synthetic spike-in standards for RNA-seq experiments. Genome res..

[15] Brown J, Pirrung M, McCue LA (2017). FQC Dashboard: integrates FastQC results into a web-based, interactive, and extensible FASTQ quality control tool. Bioinformatics.

[16] Raymond O (2018). The Rosa genome provides new insights into the domestication of modern roses. Nat genet..

